# Calcite fibre formation in modern brachiopod shells

**DOI:** 10.1038/s41598-018-36959-z

**Published:** 2019-01-24

**Authors:** Maria Simonet Roda, Erika Griesshaber, Andreas Ziegler, Ulrich Rupp, Xiaofei Yin, Daniela Henkel, Vreni Häussermann, Jürgen Laudien, Uwe Brand, Anton Eisenhauer, Antonio G. Checa, Wolfgang W. Schmahl

**Affiliations:** 1 Department of Earth and Environmental Sciences, LMU, 80333 München, Germany; 2 Central Facility for Electron Microscopy, University of Ulm, 89069 Ulm, Germany; 3 Marine Biogeochemistry/Marine Systems, GEOMAR Helmholtz Centre for Ocean Research, 24148 Kiel, Germany; 4 Pontificia Universidad Católica de Valparaíso, Facultad de Recursos Naturales, Escuela de Ciencias del Mar, Avda. Brasil, 2950 Valparaíso, Chile; 5 Huinay Scientific Field Station, Puerto Montt, Chile; 6 Alfred-Wegener-Institut Helmholtz-Zentrum für Polar- und Meeresforschung, 27568 Bremerhaven, Germany; 7 Department of Earth Sciences, Brock University, 1812 Sir Isaac Brock Way, St. Catharines, Ontario L2S 3A1 Canada; 8 Departamento de Estratigrafía y Paleontología, Facultad de Ciencias Universidad de Granada, 18071 Granada, Spain; 9 Instituto Andaluz de Ciencias de la Tierra, CSIC-Universidad de Granada, 18100 Armilla, Spain

## Abstract

The fibrous calcite layer of modern brachiopod shells is a hybrid composite material and forms a substantial part of the hard tissue. We investigated how cells of the outer mantle epithelium (OME) secrete calcite material and generate the characteristic fibre morphology and composite microstructure of the shell. We employed AFM, FE-SEM, and TEM imaging of embedded/etched, chemically fixed/decalcified and high-pressure frozen/freeze substituted samples. Calcite fibres are secreted by outer mantle epithelium (OME) cells. Biometric analysis of TEM micrographs indicates that about 50% of these cells are attached via hemidesmosomes to an extracellular organic membrane present at the proximal, convex surface of the fibres. At these sites, mineral secretion is not active. Instead, ion transport from OME cells to developing fibres occurs at regions of closest contact between cells and fibres, however only at sites where the extracellular membrane at the proximal fibre surface is not developed yet. Fibre formation requires the cooperation of several adjacent OME cells. It is a spatially and temporally changing process comprising of detachment of OME cells from the extracellular organic membrane, mineral secretion at detachment sites, termination of secretion with formation of the extracellular organic membrane, and attachment of cells via hemidesmosomes to this membrane.

## Introduction

Brachiopods are extant shell-forming, marine, sessile organisms abundant throughout the Phanerozoic, particularly during the Paleozoic when they dominated the marine benthic ecosystem. They are of interest to modern and paleo-environment research, as they cover most of the geological record and live in a wide range of marine habitats (e.g.^1–18^). Their shells consist mainly of low-Mg calcite, which is assumed to crystallize in equilibrium with seawater with only small or negligible “vital effects”.

Modern terebratulide and rhynchonellide brachiopod shells consist of up to three mineralized shell layers: the outer primary, the inner fibrous, and, where developed, an innermost columnar layer^19–22^. In two-layered shells the fibrous layer forms an extensive part of the shell. The fibres are hundreds of micrometers long and are essentially single-crystalline mineral units^23,24^. They have four surfaces: a proximal convex surface at their base, concave surfaces at their two lateral sides and a concave surface at their apical side. The shape of brachiopod fibres is unique and well developed in the Lower Cambrian, when the orders Protorthida, Orthida and Pentamerida of the class Rhynchonellata emerged with shells having fibrous microstructures^25,26^. In recent brachiopods, the morphology and dimension of fibres are characteristic for a given brachiopod species and are evolutionarily adapted to the animal’s habitat^27,28^.

Brachiopod shells are also of interest to material science, as these are important prototypes for bioinspired light-weight and energy-efficient hybrid materials. In these materials, advantageous mechanical properties of one component not only compensate for adverse properties of other’s (e.g.^29–31^), but additional gain is derived from the overall composite nature of the biological hard tissue (e.g.^32^). The mineral component provides high elastic modulus and high compressive strength, while its inferior tensile strength and brittleness is compensated by the high tensile strength and pliability of the organic matrix. The hierarchical nature of the composite hard tissue provides overall toughness and fracture toughness^33–36^.

Fibrous biological composites are an important class of materials (e.g.^29–31^). Aragonite or calcite fibres are embedded in a pliant biopolymer matrix (e.g.^34,37–45^), the latter being always cross-linked within the hard tissue (e.g.^29–31^). This enables the fibres to transmit high forces to each other via the matrix, while remaining immobile and stationary. Accordingly, in fibrous composite materials (man-made or biological) the matrix is always pliant and flexible. Biopolymer matrices are plasticized with water^30,31^, whereas, when the matrix is a mineral, the latter is always softer relative to the hardness of the constituting fibres^46^.

In biological carbonate hard-tissue the fibres are not simple rods, as it is often the case in man-made fibrous composite materials. Instead, they have highly variable lengths and thicknesses, have elaborate morphologies^27,28^ and are interleaved in three dimension^47,48^. Most biological carbonate hard tissue is subject to compressive, bending and shearing forces. As fibres within a matrix cannot be reorganized once they endure these forces, they must be properly packed and oriented within the hard tissue from the onset of their formation. This is accomplished by the formation of stacks of parallel fibres, with the stacks twisted in a plywood-like arrangement. This ensures that all components of the composite are interleaved in three dimension and on all length scales^22,48–51^.

Shell formation of brachiopods has been described based mostly on macroscopic morphological observations^25,52–54^. Williams^55–60^ investigated shell development of modern rhynchonellide and terebratulide brachiopods and postulated that the same mantle epithelium cell performs several secretory operations and is capable of secreting all shell layers. This concept is based on the proposition that mantle epithelium cells migrate during the secretionary process. As new cells are supposed to be constantly produced in the mantle groove, previously formed cells have to move away from the generative zone in a “conveyor-belt” manner. Hence, according to Williams *et al*., an individual epithelial cell secretes the periostracum first, then the calcite of the primary layer, and subsequently and in sequence, the calcite of the fibrous layer together with the organic sheath, which surrounds the calcite of the fibre^57–59^. Furthermore, due to the presumed similarity in cross-section between a fibre and the outline of a cell, Williams assumed that each cell secreted only one fibre.

Few investigations have looked at the construction of the fibre composite material of brachiopod shells by epithelial cells in any detail. Specifically, the mechanism that leads to fibre mineralization and generation of the specific morphology of a fibre is still unknown. In this study, we present the first model that describes fibre secretion as well as fibre shape formation for modern terebratulide brachiopods. We demonstrate for the terebratulide species *Magellania venosa*, (i) the very close spatial relationship of the outer mantle epithelium with the calcite fibres, (ii) the tight control of the outer mantle epithelium cells on fibre secretion, and, (iii) describe the sequence of processes that take place with brachiopod fibre formation.

## Results

**Figure 1:**
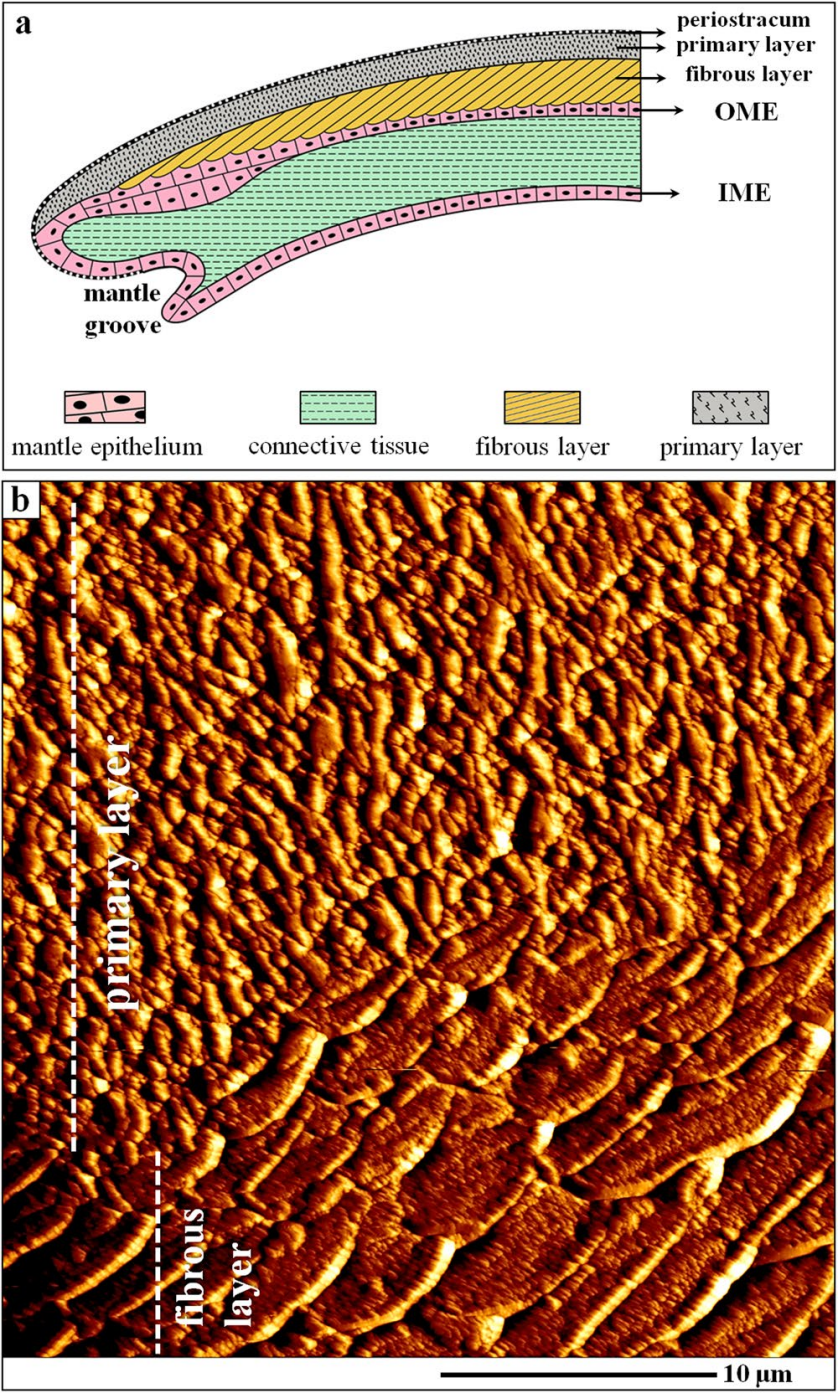
The different shell layers of the modern terebratulide brachiopod *Magellania venosa*. (**a**) Schematic deduced from our FE-SEM and TEM results depicting the position of the periostracum, the two mineralized shell layers and the location of the mantle epithelium. The schematic shows the spatial relationship between the outer (OME) and inner (IME) mantle epithelium as well as the connective tissue in the growing shell. (**b**) AFM vertical deflection image visualizing the structure of the outward primary and inward fibrous shell layer. The corresponding lateral deflection image is shown in Fig. A2a; for additional information see Fig. A1a. The fibres are sectioned transversely. Clearly visible is the transitional area between the primary and fibrous shell layers.

The scheme in Fig. 1a was deduced from our FE-SEM and TEM observations and depicts the spatial relationship between the different shell layers of the modern brachiopod *Magellania venosa* and their topological relation to the outer and inner mantle epithelia (OME, IME).

**Figure 2:**
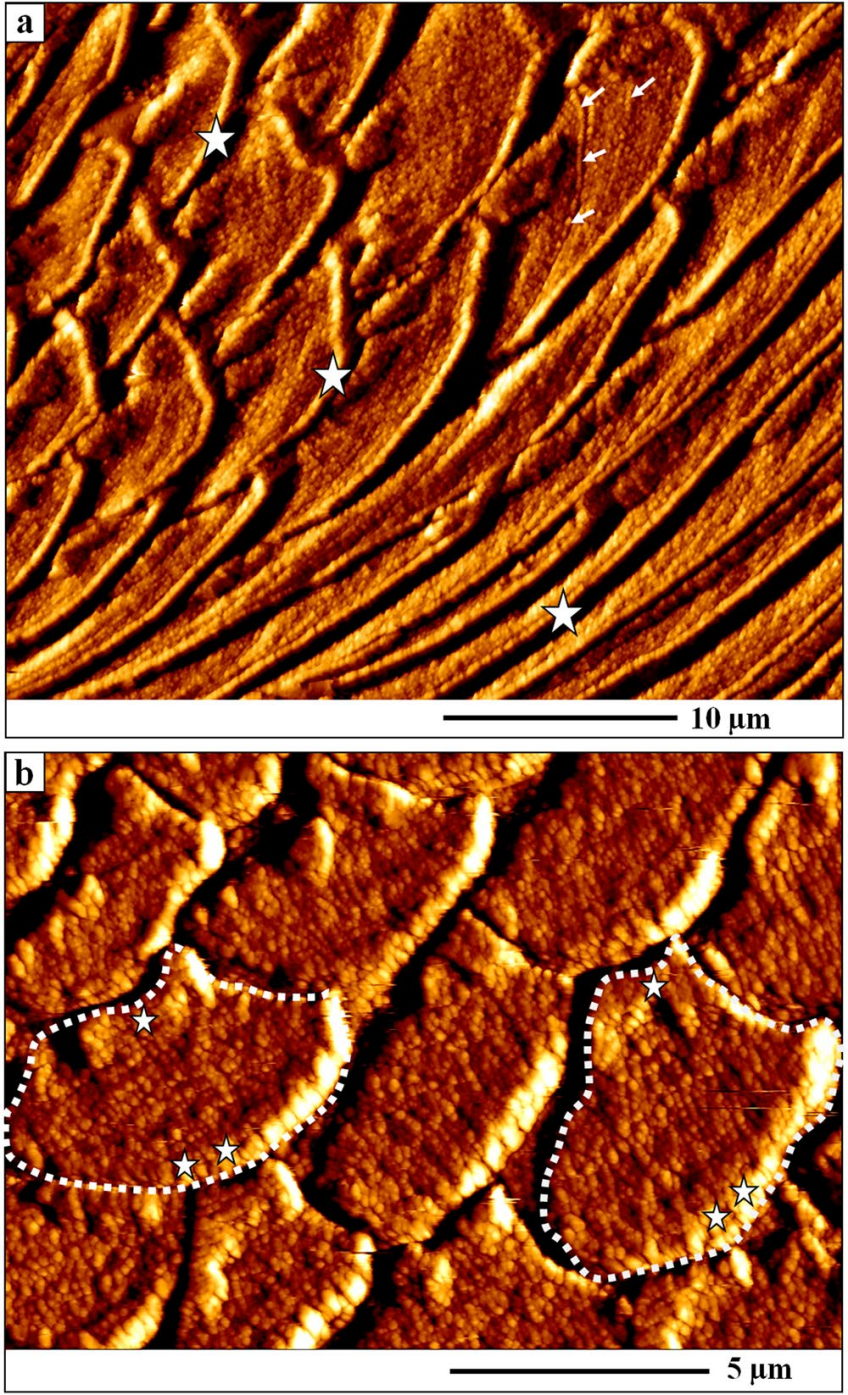
AFM vertical deflection images depicting the shape of longitudinally and transversely cut fibres as well as the internal structure of fibres of the modern brachiopod *Magellania venosa*. The corresponding lateral deflection images are shown in Fig. A2. (**a**) Nanometric internal structure of single-crystalline fibres. These nanometric units (nanometric biocalcite crystallites, NBC) are often strung in rows (white arrows in (**a**) following the convex shape of the proximal, convex basal part of a fibre and depict growth lines. White stars point to the organic membrane that lines the proximal, convex surface of fibres. (**b**) One star indicates the apical, concave part of a fibre; two stars point to the proximal, convex portion of a fibre.

**Figure 3:**
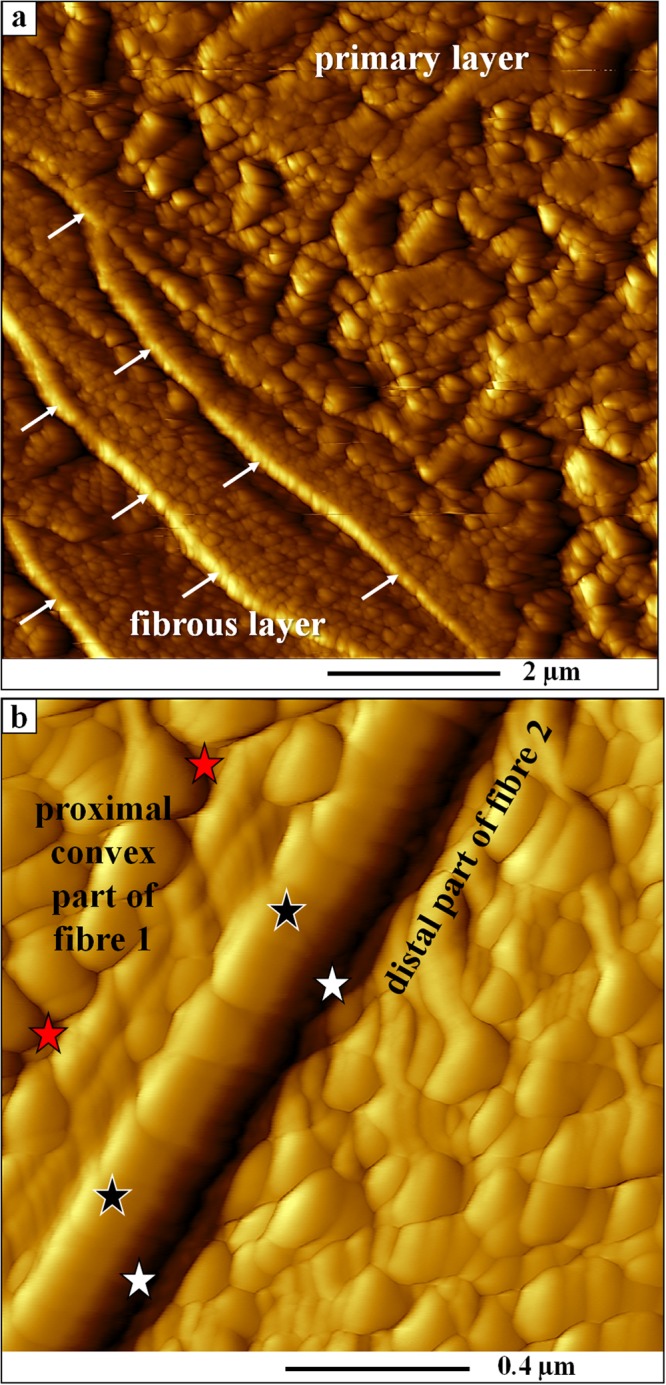
AFM vertical deflection images depicting the internal structure of primary and fibrous shell portions of *Magellania venosa*. Corresponding lateral deflection images are shown in Fig. A2. (**a**) Close-up of the primary layer and the first three rows of adjacent fibres visualizing the gradual changeover from primary to fibrous calcite shell layers. (**b**) Biopolymer membrane tightly attached to the calcite of a fibre along its proximal, convex surface. The organic membrane (black stars) is between two adjacent fibres (red and white stars) and in each case the biopolymer lines the basal (proximal), convex portion of the fibre.

The shell of *Magellania venosa* (Dixon,)^61^ consists of the periostracum, a purely organic layer, and two mineralized layers, the primary and the fibrous layer (Fig. 1a). All three layers are secreted by the outer mantle epithelium (OME) cells of the animal. The primary shell layer, located between the periostracum and the fibrous shell portion (Figs 1a,b and A1a, A2a) is secreted near the commissure and ceases to grow in thickness when the fibres (Fig. 1a) start to develop. Hence, growth of the shell in extension occurs by secretion of the primary layer at the commissure, while growth in shell thickness takes mainly place with secretion of fibres some tens of micrometers away from the commissure (Fig. 1a). The fibres (Figs 1b, 2 and A1b, A2) in *Magellania venosa* have four sides: one convex side facing proximally, two concave sides facing laterally and one concave side facing distally. The fibres are separated from each other by an organic membrane (Figs 3 and A1a, A1b, A2f), but this membrane does not form a sheath around individual calcite fibre’s. Instead, the membrane lines only the proximal, convex surface of a fibre (Figs 3 and A1b, A2f). The specific shape and mode of packing of the fibres implicates the full encasing of the calcite of a fibre by organic substance.

AFM images (Figs 1b, 3a and A1a, A2d) visualize the transition from the primary to the fibrous shell layer. There is no distinct or sharp boundary between the primary layer calcite and the adjacent secondary-layer fibres but rather a smooth transition (Figs 3a and A1a, A2d). Mineral units that are next to or close to the primary layer portion of the shell do not show yet the characteristic blade-shaped morphology of a brachiopod fibre (white stars in Fig. A4b). Instead, they are rather irregular in shape and elongated in cross-section. They are, however, already lined along their proximal, convex side by an organic membrane (white arrows in Figs 3a and A2d). Occasionally short segments of organic membrane might become visible within the transition zone between the primary and fibrous shell layers (white arrows in Figs A4a and A4b). Some distance away from this transition region, fibre morphology becomes more regular and cross-sections of fibres increase in size (yellow stars in Fig. A4b). The calcite within fibres is crystallographically highly aligned like a single crystal^22–24^. Upon etching it displays an internal nanometric structure (nanometric biocalcite crystallites (NBC), Figs 2 and 3, A2, A3). The internal structure often displays curved rows, growth lines, which follow the convex proximal surface of the fibre (white arrows in Fig. 2a).

**Figure 4:**
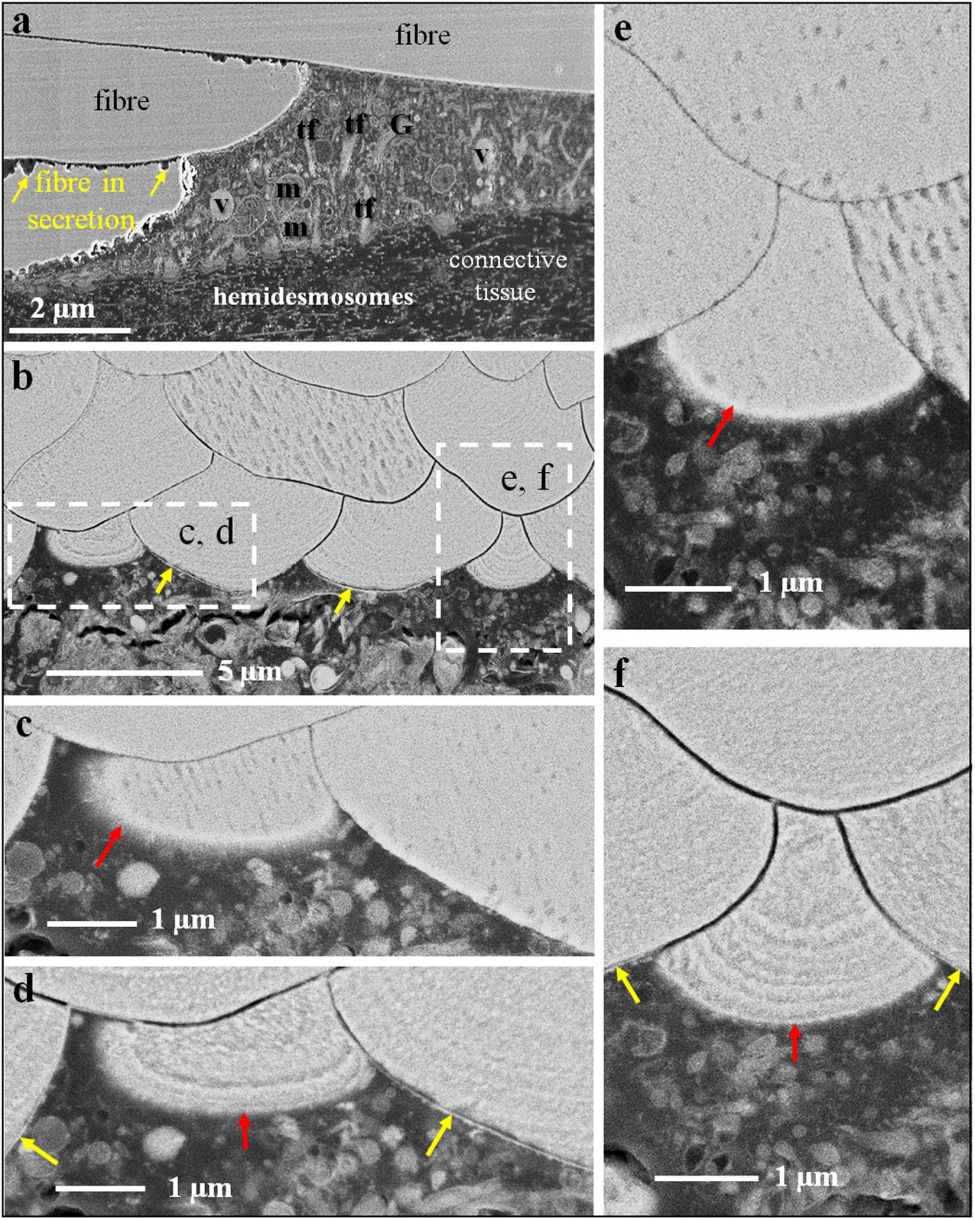
FE-SEM micrographs of polished surfaces of chemically fixed (**a**), high-pressure frozen and freeze-substituted (**b**–**f**) and etched (**d**,**f**) shell pieces of *Magellania venosa*. Samples in (**b**,**d**,**f**) were etched at a pH of 9, for 40 seconds with a 0.1 molar HEPES and 2,5% glutaraldehyde solution and critical point dried. Samples in (**c**,**e**) were polished but not etched nor critical point dried. Micrographs were recorded using secondary electron (at 4 kV; **a**) and converted backscattered electron (at 4 kV; **d**–**f**) signals, respectively. (**a**–**f**) Outer mantle epithelium (OME) cells are always in very close contact to the calcite of the fibres. It is well visible that at some cell – calcite interfaces the extracellular biopolymer lining of the fibre is not developed (red arrows in **c,e,d,f**). These are the sites where fibre formation is still in progress. In contrast, at sites where the extracellular biopolymer membrane along the proximal, convex surface of a fibre is well observable (yellow arrows in **b,d,f**), fibre mineralization is complete. At their basal side, epithelial cells are connected to the connective tissue by basal hemidesmosomes (red dots in **a**). Samples (**c**,**d**) are high-pressure frozen, freeze-substituted, embedded in EPON resin and polished with a diamond knife; samples (**d**,**f**) are, in addition, etched for possible detection and visualization of amorphous calcium carbonate. G: Golgi apparatus, m: mitochondria, t: tonofilaments, v- vesicles.

Figure 4 shows FE-SEM micrographs of polished surfaces of chemically fixed (Fig. 4a) and high-pressure frozen and freeze-substituted (Fig. 4b–f) shell portions embedded in EPON resin. High pressure freezing followed by freeze-substitution in acetone containing OsO_4_ and uranyl acetate ensures minimal shrinkage of the soft tissue and negligible dissolution of the calcite during preparation. We find that the outer mantle epithelium is always in close contact with the proximal, convex side of the fibres. On the basal side, mantle epithelium cells are connected to the basal lamina of the connective tissue by large hemidesmosomes (red dots in Fig. 4a). In high pressure frozen and freeze-substituted samples, at sites of mineral secretion, apical cell membrane cannot be distinguished from basal surfaces of fibres (Fig. 4c,e). This indicates that in *Magellania venosa* extrapallial space between fibres and OME cells is either absent or only very few nanometers wide.

As Fig. 4c,e visualize, in *unetched* samples, the organic membrane that lines the proximal, convex side of a fibre cannot be distinguished from the calcite of the fibre. However, when *etched*, the membrane becomes visible (yellow arrows in Fig. 4b,d,f). Hence, there is a close connection between the membrane that lines the calcite of the fibres, in that this organic membrane is an integral part of fibres. The close connection between fibre calcite and membrane lining is also clearly visible in our AFM images (Fig. 3b; black and red stars at the basal, convex surface of a fibre).

In *unetched* samples (Fig. 4c,e) the organic membrane is not visible in FE-SEM images as during freeze-substitution OsO_4_ and uranyl acetate have no access to them. In *etched* samples, we find between the organic membrane lining at the proximal, convex part of a fibre and the distal section of the adjacent concave fibre surface a recess (Figs 3b, 4e,c and A2). This might be due to higher solubility of the mineral at distal fibre surfaces and can be explained by inhibition of calcite crystal growth at these sites. This leads to the formation of nanocrystalline calcite with higher solubility.

In *chemically-fixed* samples with fibres being still in formation, we observe irregular dissolution of the calcite at fibre margins (yellow arrows in Fig. 4a). However, in *high pressure frozen and freeze-substituted* samples these dissolution features are not present (Fig. 4c,e). Accordingly, etching of high pressure frozen and freeze-substituted shell portions with an aqueous solution at a pH of 9 containing 0.1 molar HEPES and 2,5% glutaraldehyde does not result in dissolution of the mineral (amorphous or crystalline) of the developing fibres. Hence, the dissolution features that we observed in the chemically fixed samples (Fig. 4a) can be traced back to the effect of aqueous solutions that were used in the course of that preparation method. They do not indicate a possible presence of an amorphous precursor, amorphous calcium carbonate (ACC), within the fibres. Furthermore, we do not find selective dissolution of the calcite between epithelial cells and at the sites of mineral secretion (red arrows in Fig. 4d,f). As ACC readily dissolves at a pH of 9, this is a good indication that the calcite of fibres forms directly and most probably not via a disordered mineral phase, such as amorphous calcium carbonate.

**Figure 5:**
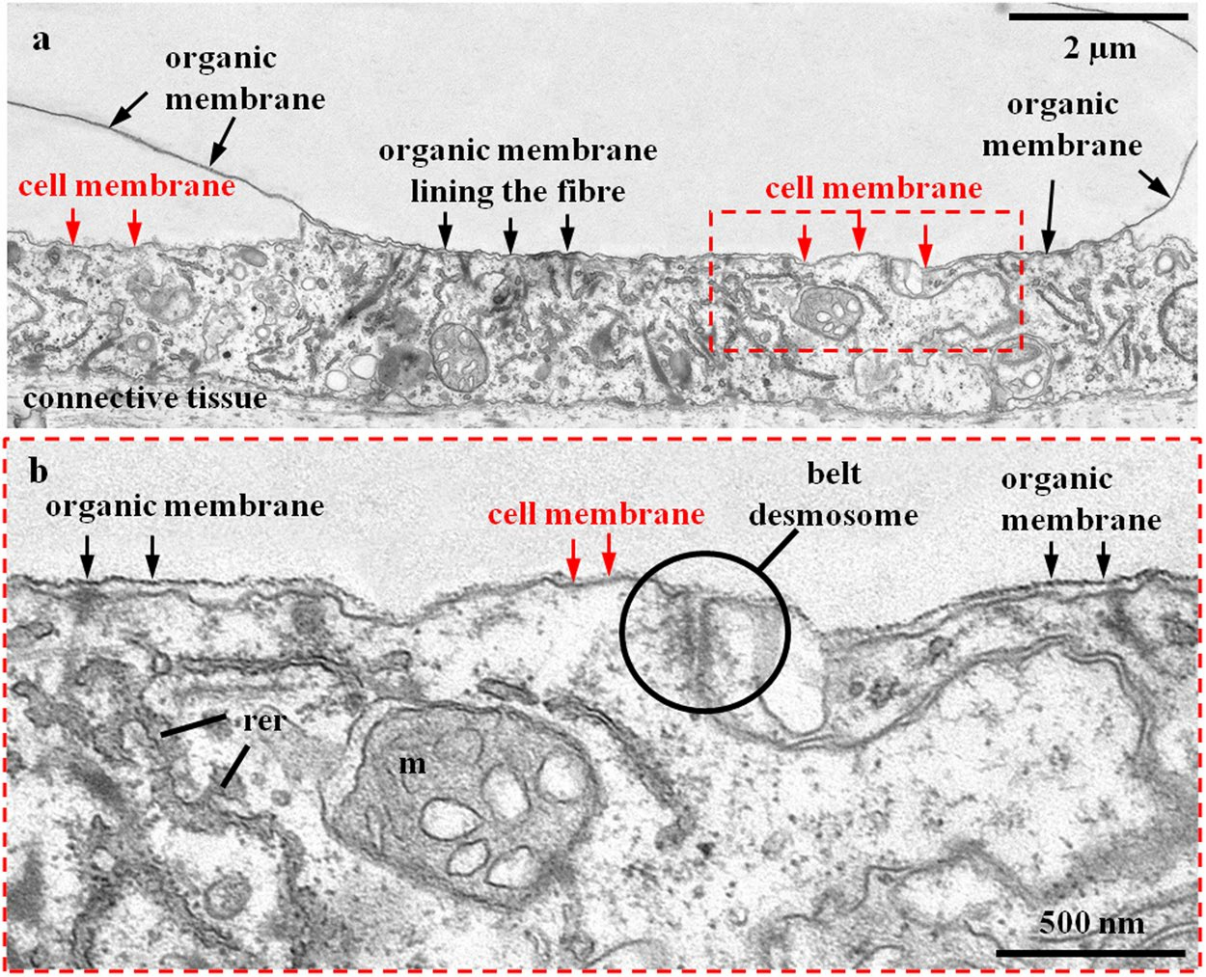
TEM micrographs of chemically fixed and decalcified contact between epithelium and shell calcite in modern *Magellania venosa*. (**a**) Mineral formation ceases with the secretion of an organic membrane covering the proximal, convex part of the fibre (black arrows). At these sites, we see two membranes: (i) the apical cell membrane of the attaching OME cell (red arrows), and (ii) the organic membrane lining the basal portion of the fibre (black arrows in b). (**b**) Site of active fibre secretion, there is only one membrane present and visible, namely the apical membrane of the OME cell (red arrows in **a** and **b**), which is tightly attached to the calcite of the forming fibre. Neighbouring cells are connected to each other by belt desmosomes. Note the absence of tonofilaments in cells below those parts of the fibre that are actively secreting. rer: rough endoplasmatic reticulum, m: mitochondria.

**Figure 6:**
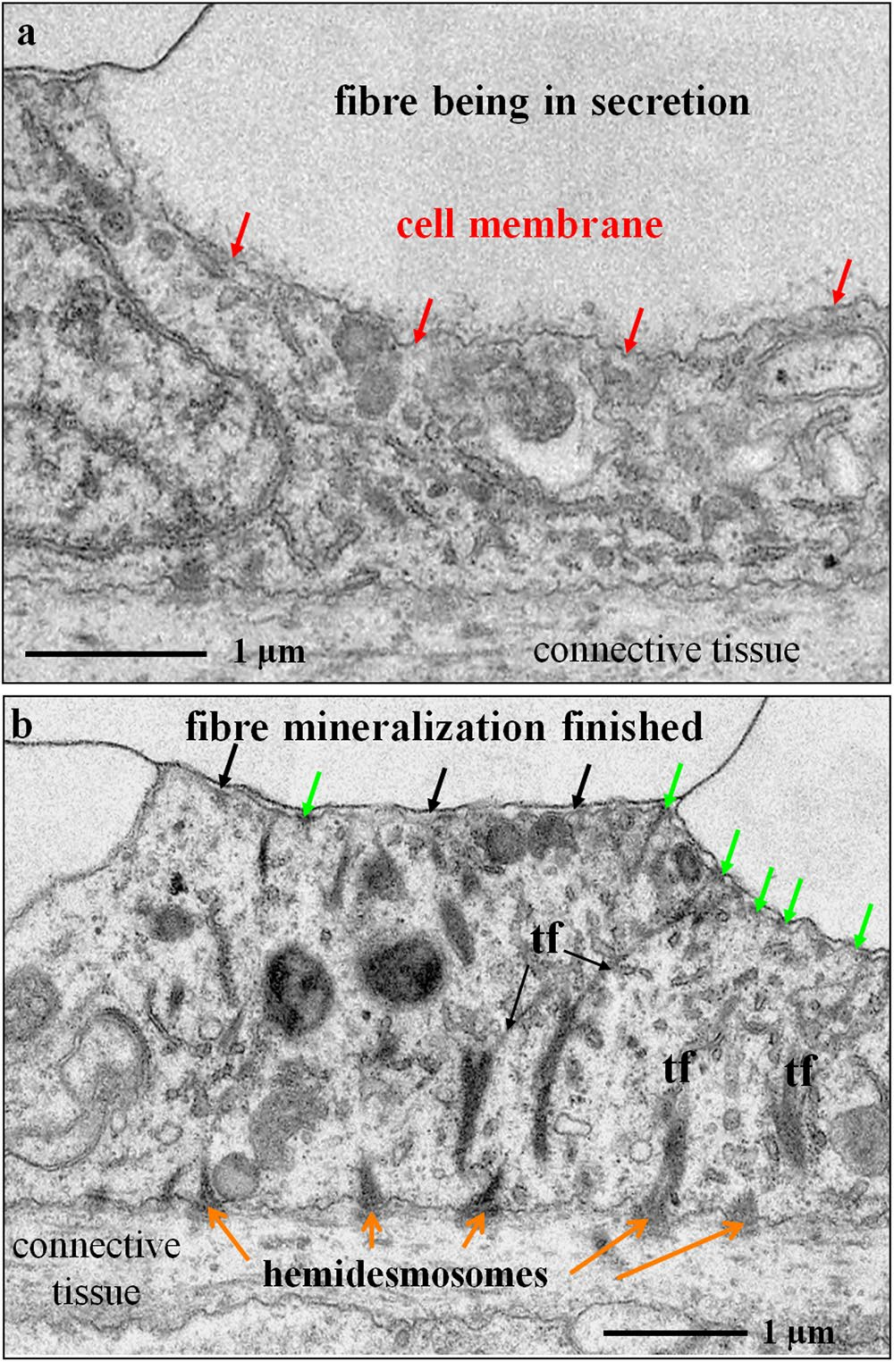
TEM micrographs of chemically fixed and decalcified contact between epithelium and fibre calcite in modern *Magellania venosa*. Samples were taken from the central region of the shell. (**a**) With ongoing mineralization, the membrane lining the proximal, convex part of the fibre is not yet developed (red arrows). (**b**) Apical cell membrane attached to organic membranes of the fibres by apical hemidesmosomes (green arrows), the latter being connected to basal hemidesmosomes (orange arrows) via tonofilaments (tf). Cells below fibres in the process of active mineral secretion do not contain any tonofilaments.

**Figure 7:**
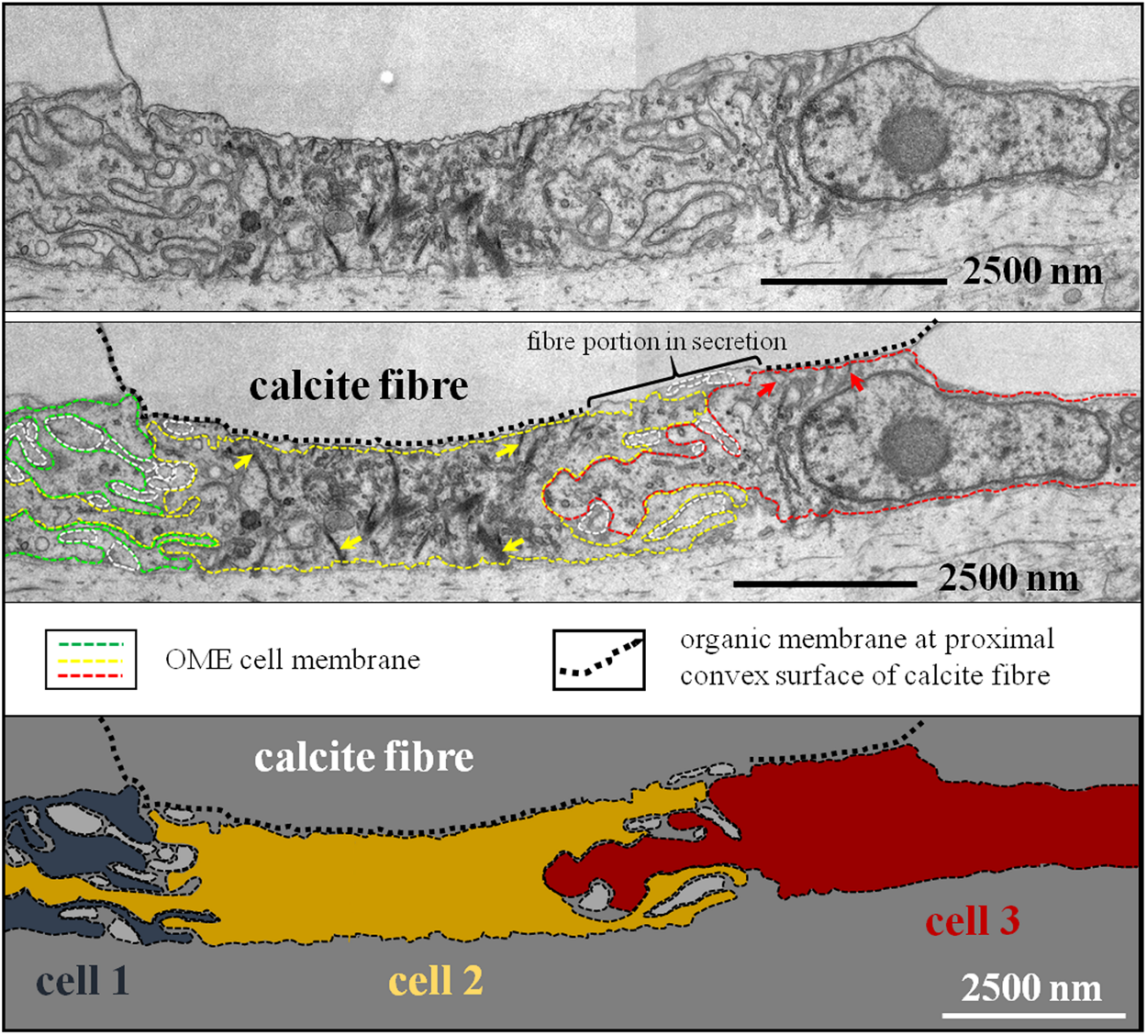
TEM micrographs and deduced schematic showing the interlinkage of three cells below an almost fully secreted fibre. Well visible are tonofilaments within cells 2 and 3 (yellow and red arrows) that connect the apical cell membrane to the organic membrane at the proximal, convex side of the fibre by hemidesmosomes.

TEM imaging of chemically fixed and decalcified shell samples (Figs 5–7) allows us to investigate the ultrastructure of OME cells, the organelle distribution within them, and the topological relation of OME cells to the organic membrane that lines the proximal, convex surface of adjacent fibres.

Apical membranes of OME cells are always present in the investigated samples, in contrast to membrane that lines the basal (proximal), convex surface of fibres. We find regions where the organic membrane lining at the basal (proximal) surface of the fibre is lacking (red arrows in Fig. 6a), is incomplete (Figs 5 and 7), or is fully developed (black arrows in Fig. 6b). When both membranes are present, apical cell and organic membranes, at the basal surface of the fibres, OME cells are attached to the organic membrane of the fibres via apical hemidesmosomes (green arrows in Fig. 6b). At sites where the organic membrane at the proximal, convex surface of the fibre is lacking, OME cells do not contain any hemidesmosomes or tonofilaments (Figs 5b and 6a). At these sites, mineral transport from OME cells to adjacent fibres, thus active secretion, is still in progress. Analysing an epithelial length of 189 µm we find that 14 regions with a total length of 98 µm are attached to the shell via hemidesmosomes and 10 regions with a total length of 91 µm are not (Fig. A5-c). Thus, at a given time, about 52% of the OME is not secreting mineral and only a maximum of about 48% of the OME is involved in fibre mineralization.

Contrasting to observations by Williams and co-workers^25,56–60^, we do not find a one-by-one relationship between epithelial cells and fibres. Instead, we often observe either one cell below two or more fibres or interdigitating epithelial cells below one fibre (Fig. 7). In regions where the organic membrane at the proximal, convex side of the fibre is not fully developed, cross sections through fibres reveal that secretion of the extracellular organic membrane starts at the two lateral edges (corners in the two-dimensional cross-section) of the fibre (Fig. 5a) and progresses from here until the proximal, convex surface of the fibre is fully covered with the organic lining. The formation of the basal membrane lining at the convex side of the fibre represents the terminal step in fibre secretion. It also separates the outer mantle epithelium cell from the compartment in which the fibre is mineralized. Where the fibre basal membrane is absent, the compartment in which calcite mineralization takes place is in direct contact with cell membranes, such that either by pumps or ion exchange mineral components can be transported into the compartment of calcite crystallization.

## Discussion

### Fibre secretion and microstructure generation in *Magellania venosa*

Elongated, cylindrical mineral units are structural elements in bivalve, gastropod and brachiopod shells and are either prismatic-columnar, foliated, fibrous or acicular in shape (e.g.^62–64^). Even though prismatic-columnar, foliated and acicular microstructures prevail, in some classes of the phyla Mollusca and Brachiopoda assemblies of fibres are of major importance to the overall structure of the hard tissue and may constitute entire shell layers. Fibrous microstructures prevail in the shell of Mytiloida and Cavolinioidea (Mollusca) and in Rhynchonellata and Terebratulida (Brachiopoda). In most cases fibres are made of calcite (with the exception of the shells of the marine cavolinioidean gastropods, where they are made of aragonite) and vary in shape and dimension depending on the taxa.

Rudwick^52,^^53^, Rosenberg^65^ and Rowel and Grant^66^ described shell architecture and growth in modern and fossil brachiopods. Williams and co-workers^25,56–60^ investigated brachiopod shell mineralization and hypothesized from SEM and TEM observations that (i) the same cell of the outer mantle epithelium lobe is able to perform different secretory tasks and secretes sequentially all layers of the shell, (ii) based on similar cross-sections between fibre and outline of the cell, a fibre is formed by one cell only, and (iii) each fibre is entirely encased by an organic membrane.

In our study, we did not observe any features to support these findings. Instead, we observed that near the commissure, the OME consists of many cell layers, while, further away towards central shell portions, OME cells form a single layer (for a detailed study see^67^). We did not find any similarities in cross-section dimensions between cells and fibres. Cross sections of OME cells varied and we observed roundish as well as elongated cross-sections of mantle epithelial cells^67^. Furthermore, we found that neighbouring cells, each of them attached to the same fibre cooperate in fibre secretion^67^ (Fig. 7 this study and^67^). We observed that epithelial cells are only in contact with the proximal, convex side of the fibre and never in contact with their concave sides (not even in puncta). Thus, the membrane, that is formed in the final step of fibre secretion, is exclusively deposited onto the basal, convex surface of a fibre. It is the matrix membrane located between fibres, or the extracellular matrix within the shell (Figs 5a, 6b and A1b, A2f, A3a). The interlocked packing of fibres with their concave-convex morphology leads to the perception that each fibre is sheathed by an individual membrane. In modern brachiopod shells, only one of the four surfaces of any individual fibre is lined by an organic membrane.

Most biological as well as bioinspired structural materials are composites of soft and hard components. They consist of a soft polymer scaffold that is reinforced by hard minerals (in the case of biological hard tissue) or/and ceramics (in the case of biomimetic/bioinspired hard materials). Even though biological and biomimetic hard tissues share this basic material property, their mode of fabrication is quite distinct. Synthetic composites formed by freeze-casting have structures, architectures and even material properties that are to some extent comparable to those of biological composites^68–70^. However, a basic difference unique to biologic composites is that fabrication of synthetic composites occurs in at least two steps. First, the scaffold is formed and, in a subsequent step, the scaffold is reinforced with another material^68,70^ (Fig. 1 in^68^, Fig. 5 in^70^). The generation of biological hard tissue follows a different pathway. It is a layer-by-layer formational process comprising the sequential deposition of mineral and, when and where needed, secretion of a biopolymer membrane, or vice versa. The latter are, for example, the polymer lining at the convex surface of a brachiopod fibre at termination of fibre formation (this study) or an interlamellar or surface membrane during molluscan nacre growth^71,72^.

When brachiopod fibre and nacreous tablet formation are compared, significant differences emerge in biopolymer/mineral deposition and, hence, microstructure generation. In the case of modern brachiopod fibres, during secretion, mantle epithelium cells are always in direct contact with the mineral (this study), whereas in molluscs the nacreous tablets are never in direct contact with epithelial cells. There is always an interlamellar (in bivalves) or surface (in gastropods) membrane between secreting mantle cells and the growing aragonite platelets^72^ (and references therein). In bivalve nacre, the aragonite is always deposited between a few (two or three), and in gastropod nacre even between many (a few tens) interlamellar membranes (Fig. 7A,B,F,G in^72^). When brachiopod fibres form, secretion of the biopolymer membrane covering the convex surface of the fibre is the last and *terminal step in fibre growth*. In contrast, when nacre forms, *aragonite tablet formation is started* with the consecutive self-assembly by liquid crystallization of interlamellar membranes. This leads to the formation of compartments that become successively infiltrated along mineral bridges by aragonite and ultimately filled with nacreous tablets (e.g. Fig. 7A,F,C in^72^ and references therein). Accordingly, we find modern brachiopod shell and molluscan nacre development as two divergent microstructure generation processes (Fig. A7a and^72^). One is biologically controlled through direct cellular contact and activity with the mineral as it is the case for brachiopods, the other is physically controlled through the self-organization of extracellular matrix membranes as it is the case for molluscan nacre (Fig. A7a and^72^).

In summary, in many man-made biomimetic composites the eventually mineralized organic matrix is fully developed prior to mineral infiltration. Formation of molluscan nacre resembles to some extent the formation of biomimetic composites, as it occurs through mineralization of a preformed biopolymer matrix. However, nacre growth is a dynamic process, the mineralization front advances with ongoing shell growth. During shell growth, extracellular matrix formation progresses steadily and it is successively mineralized. Brachiopod fibre formation is a strictly layer-by-layer deposition process, where both, secretion of the mineral and the biopolymer is controlled synchronously by mantle epithelial cells (Fig. A7a).

### Fibre shape generation in *Magellania venosa*

Brachiopod fibrous layer microstructure, such as fibre morphology and their arrangement in stacks, is characteristic of modern terebratulide and rhynchonellide brachiopod shells (see the compilation of^27,28^). It differs from that in other biological hard tissues, for example, calcite fibres in *Mytilus edulis* shells (Fig. A6). In the latter, fibre shape is more cylindrical and the mode of interlocking is less regular (Figure A6b)^73^. The mode of assembly of fibres in modern brachiopod shells shows similarities in cross-section to the “brick-wall” arrangement of nacreous tablets in bivalve nacre (Fig. A7b). Hence, the staggered organization of basic mineral units, irrespective of these being tablets or fibres, is obviously a type of microstructure that is of high value to many shelled organisms and was and is utilized in very different aquatic habitats. Furthermore, it was developed within the geologic record by many organisms of different phylae. Hence, basic mineral unit (fibre, tablet) morphology and mode of interlinkage is essential to the organism, as an adequately constructed shell guaranties protection of the soft tissue and, thus, survival of the organism in its chosen habitat (e.g.^74^).

**Figure 8:**
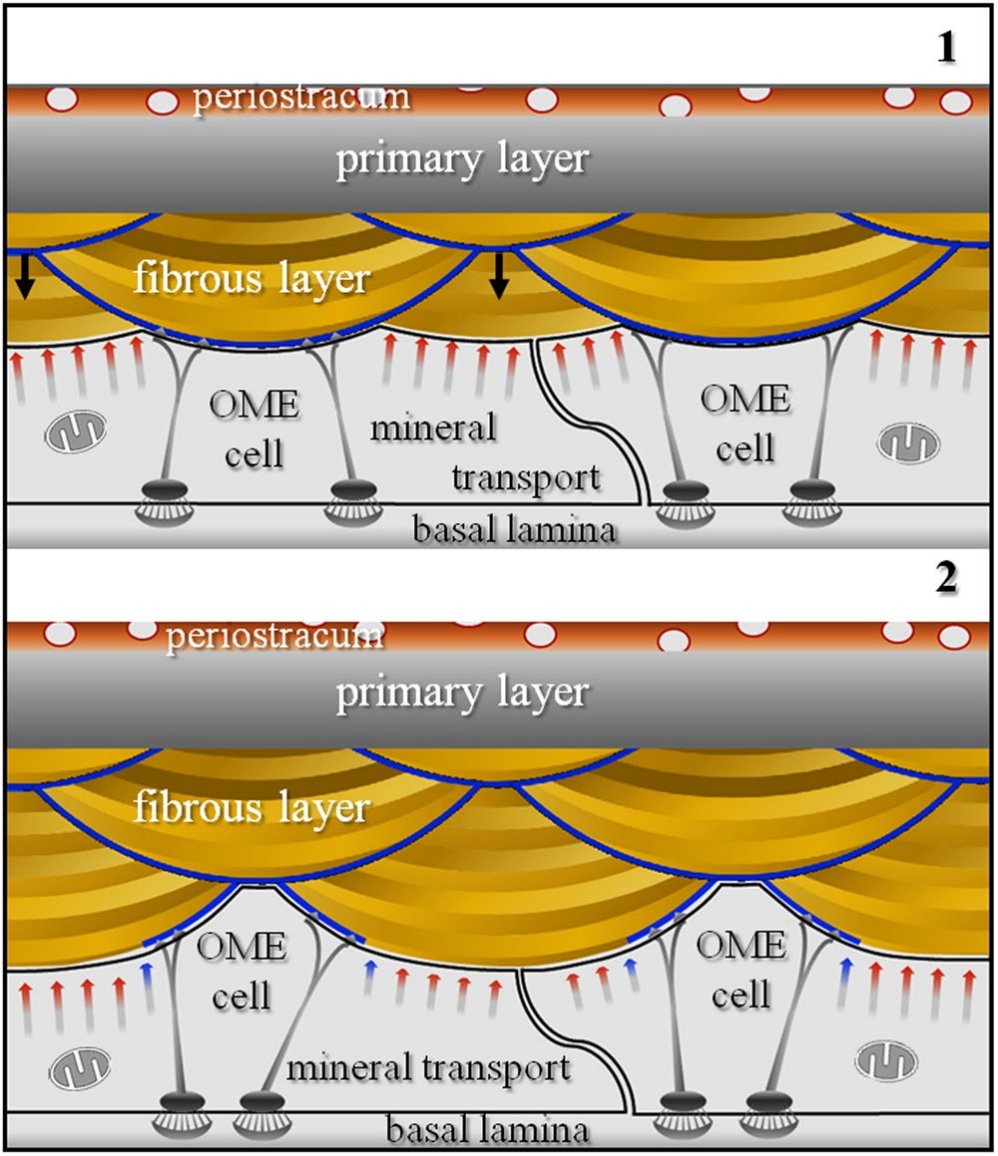
Schematic model illustrating calcite fibre shape formation for terebratulide and possibly rhynchonellide brachiopods. We see a stack of transversely cut fibres. Prior to fibre secretion OME cell membranes are in close contact with the extracellular organic membrane lining present along the proximal surface of fibres. Detachment of epithelial cells from this membrane lining induces mineral secretion and starts fibre growth (black arrows in schematic 1). When fibres have reached their full width, OME cells start to secrete the organic membrane lining (blue arrows in schematic 2), and when finished, will completely line the basal convex part of the fibre (blue stars in schematic 2).

**Figure 9:**
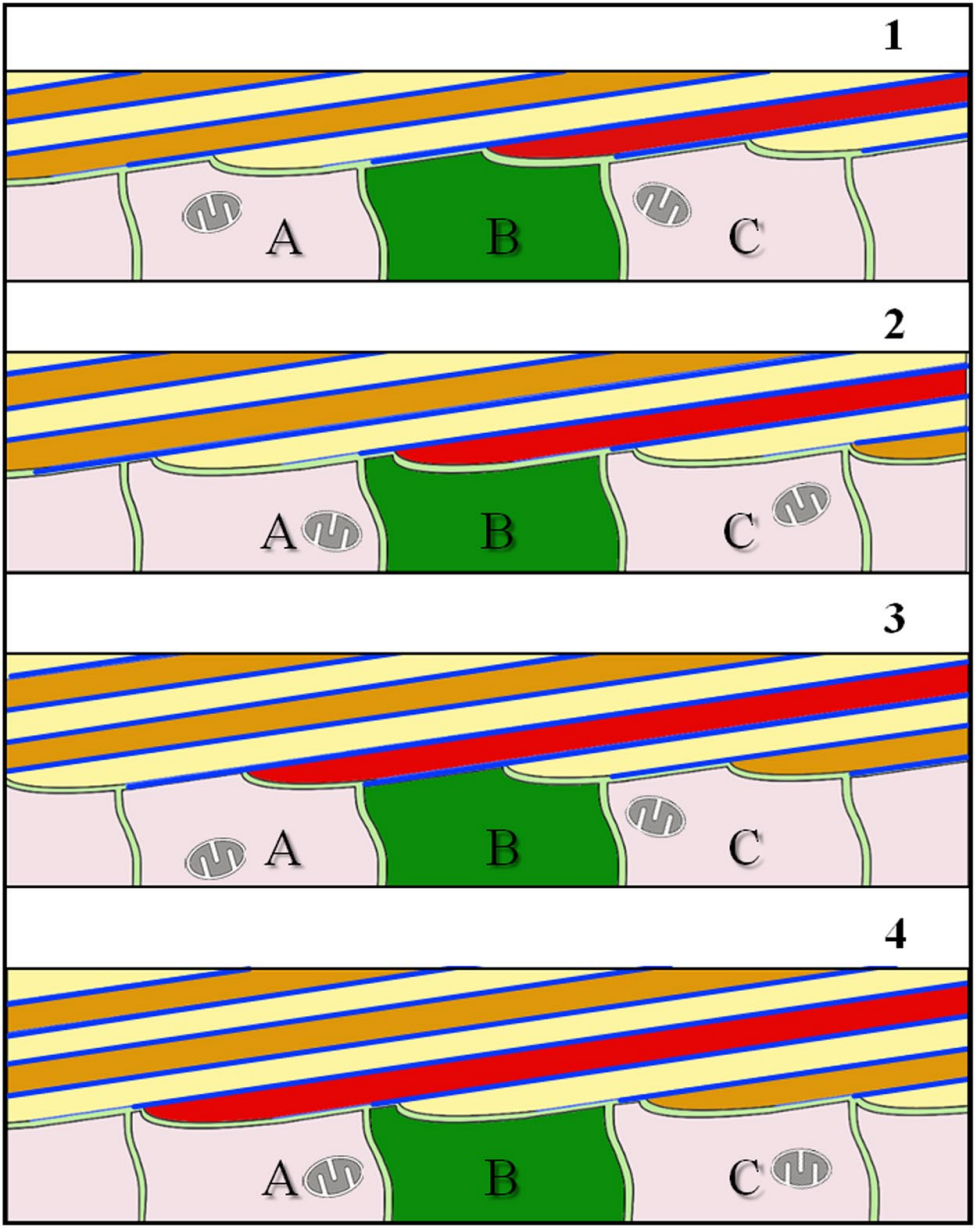
Schematic model illustrating calcite fibre elongation for terebratulide and rhynchonellide brachiopods. A stack of longitudinally cut fibres is shown. Fibre growth occurs through the coordinated interaction of neighbouring cells (A to C). These are stationary and secrete both, the organic basal lining as well as the calcite of the fibre, and this in the required proportion necessary for the developing fibre (stages 1 to 4). Elongation of fibres takes place by repeated continuous changes in the position of growing fibres relative to cells: (i) attached either to the organic membrane lining the convex surface of the fibre or (ii) to the calcite of the fibre. The organic membrane lining the fibre is indicated with blue lines. Due to the absence of a one-by-one relationship between epithelial cells and calcite fibres, as the shell grows in thickness, each epithelial cell contributes to the formation of more than one fibre and secretes both calcium carbonate and organic material at different portions.

Observations on the unique morphology of brachiopod fibres led us to develop a model for fibre shape generation (Fig. 8) and fibre elongation (Fig. 9) for the shell of *Magellania venosa*. This model may be applicable to other modern and fossil terebratulide and rhynchonellide brachiopod species that produce a shell layer with a fibrous microstructure. Our model is based on the following observations: (i) only about 50% of the epithelium secretes mineral at any given time as the remaining part of the epithelium is tightly attached to the shell via hemidesmosomes and therefore cannot secrete any mineral. (ii) The extrapallial space either does not exist or is very narrow what indicates that mineral secretion is under tight cellular control. (iii) At large epithelial lengths, sites of mineral secretion alternate with sites that do not secrete any mineral. (iv) Only the convex part of any individual fibre is covered with an organic membrane. (v) Secretion of the organic lining of the proximal convex surface of the fibre proceeds from the sides to its central part.

Fibre formation starts with the disintegration of apical hemidesmosomes and the detachment of a small region of the outer mantle epithelium from the organic membrane lining of a previously secreted and finished portion of a fibre (black arrows in Fig. 8, sketch 1). This induces mineral accretion at this site by the underlying cell or cells. In cross section, this detached region appears to be small at the beginning. However, it increases in size and thickness with progressive fibre growth (Fig. 8, sketch 1). Once the fibre has reached its full width, the underlining epithelial cells start with the secretion of the membrane that lines the basal surface of the fibre. The process starts at the edges (corners in the two-dimensional cross-section) of the growing fibre (blue arrows in Fig. 8, sketch 2). With ongoing secretion, the proximal convex surface of the growing fibre is lined with a membrane until full coverage is achieved of the convex fibre surface (blue stars in Fig. 8, sketch 2). Once the latter is completed, apical OME cell membrane attaches itself immediately via apical hemidesmosomes to extracellular organic membranes at the proximal side of the fibres (Fig. 8). In between fully secreted fibres, epithelial cell membranes are still in direct contact with the calcite of the fibre and carry on with mineral secretion of these (not yet finished) fibres (Figs 5a, 7 and 8, sketch 2). The attachment of mantle epithelia to extracellular membrane portions at the proximal convex surface of the fibres is essential for stabilization of the whole secretion system. This is important for both, for fully developed fibres and fibres that are still actively in the secretion process.

Additional proof for the above described model of fibre growth was observed in AFM images. The striation patterns that we find on cross-sections of transversely and longitudinally cut fibres (white arrows in Fig. 2a) supports the incremental addition of mineral to the growing fibre by successively retreating OME cells. Mineral secretion ceases when the organic membrane forming at the two edges (corners in the two-dimensional cross-section) of a fibre merges (Figs 5, 7 and 8 sketch 2), and the membrane lining fully covers the proximal, convex fibre surface.

We did not observe a one-to-one relationship between epithelial cells and calcite fibres, hence, each epithelial cell may contribute to the formation of many fibres when the shell grows in thickness. Figure 9 shows a model for fibre elongation and depicts a sequence of four moments in time (Fig. 9, sketches 1–4). The model emphasises how individual fibres are formed by many cells with each cell being engaged in the secretion of just a short segment of a fibre (e.g., Fig. A5). We depict three individual cells (A–C in Fig. 9) contributing from right to left to the elongation of three different fibres. In our model, individual epithelial cells do not need to move along the inner shell surface, nor does our model imply sliding of the cells as fibres grow across the shell. Instead, elongation of fibres is brought about by repeated changes in sites where (i) regions of a cell secrete the organic membrane at the proximal, convex surface of a fibre (blue lines in Fig. 9), (ii) stay attached to it and (iii) elongate the fibre by mineral secretion after detachment from the organic membrane. This can also easily explain why the brachiopod fibre calcite composite is produced in a plywood-like manner, where, in stacks of parallel aligned fibres, the direction of the morphological fibre axis is changed. This is difficult or impossible to envisage with the ‘conveyor belt’ model of Williams^56–58,60^.

In summary, when the shell grows in thickness, each epithelial cell contributes to the formation of many fibres and cooperates with its neighbours. In the native state, cells assume a near hexagonal pattern, so that each cell has to cooperate with up to 6 neighbouring cells and, thus each cell is able to contribute to the formation of more than two fibres at a given time. Furthermore, each cell can secrete simultaneously calcium carbonate as well as biopolymers, hence, there are areas within the same cell that secrete calcium carbonate, while other regions of that cell produce organic material. In addition, the extent of these carbonate and biopolymer secreting regions of the cell changes with time at the secretion of brachiopod fibres.

## Conclusions

Our results show that fibre secretion and fibre shape formation in the modern brachiopod *Magellania venosa* is a dynamic process. It requires a sequence of actions induced and coordinated by outer mantle epithelium cells (OME) in close contact with the forming fibre.

We reach the following conclusions for the development of calcite fibres and extracellular organic matrix membranes in the shell of the modern brachiopod *Magellania venosa*:

1. It is shown for the first time that extrapallial space between the fibres and the outer mantle epithelium is either non-existent or is extremely narrow and only a few nanometres wide. This indicates that fibre formation in *Magellania venosa* is under tight cellular contact and cellular control. Our results do not support mineral transport from the cell by organelles (such as vesicles), but rather indicate that cells secrete calcium and carbonate ions via ion transport mechanisms in the cell membrane.
2. Frequently more than one cell contributes to the formation of a calcite fibre at the same time; hence, fibre secretion, growth and shape generation requires communication of adjacent OME cells.
3. The extracellular organic membrane is secreted only onto the proximal, convex surface of a fibre.
4. Fibres are not individually and completely sheathed by separate organic membranes.
5. Secretion of calcite by epithelial cells occurs only at sites where the extracellular organic membrane at the proximal, convex surface of the fibre is absent.
6. Once the extracellular membrane at the base of fibres is secreted, cells of the outer mantle epithelium are attached to these by apical hemidesmosomes. This keeps the OME close to the shell and stabilizes those fibre regions that are still in active secretion.
7. A brachiopod fibre is formed by a cyclic sequence of processes occurring at the apical OME cell membrane: (i) local detachment of epithelial cell membrane from the extracellular organic membrane of previously formed fibres, (ii) resumption of secretion of calcite at these detachment sites, (iii) once the final width of the fibre is reached, secretion of an extracellular membrane starting from the edges of the fibre and progressing towards the centre of the convex fibre surface; mineral secretion is suspended where this extracellular membrane is formed, (iv) attachment of the cells via apical hemidesmosomes to the newly formed extracellular organic membrane.
8. Calcite secretion is suspended where the proximal, organic membrane terminating the secretion of the fibre is formed, and it is resumed in locations where OME cells detach from the extracellular membrane of a previously finished fibre by resorption of the hemidesmosomes.
9. The fibrous layer of rhynchonelliform brachiopod shells is a hybrid fibre composite material that has an overall plywood-like organization with the basic mineral units, the fibres, being assembled in a microstructure resembling the ‘anvil-type’ arrangement of calcite fibres in bivalves. The microstructure also compares to the “brick wall’ arrangement of aragonite tablets in bivalve nacre, but rather than being simple “bricks”, the mineral units are fibres with the characteristic cross section generated by the process of simultaneous growth of parallel fibres.

## Materials

We investigated fibre shell formation of the modern brachiopod *Magellania venosa* from Patagonian Comau Fjord (southern Chile). Brachiopods were collected by scientific SCUBA divers at about 21 m water depth, average water temperature was 11 °C and salinity 30.3. Samples that were chemically fixed and decalcified had a longitudinal axis length between 5 and 7 mm; shells that were fixed by high pressure freezing and subsequent freeze substitution had a longitudinal axis length of about 4–5 mm.

### Sample preparation

#### Chemical fixation and decalcification

A total of 8 small samples of the shell with the mantle tissue attached were first dissected from central and commissure regions of both ventral and dorsal valves. We used two different fixation media. Fixation medium A was prepared by mixing equal volumes of filtered seawater from the culture of *Magellania venosa* containing 2% paraformaldehyde and 2% glutaraldehyde with a solution of 0.35 mol L^−1^ saccharose and 0.17 mol L^−1^ NaCl in 0.2 mol L^−1^ Na-cacodylate buffer (pH 7.7). Fixation medium B was prepared in the same way, however, with 3.2% paraformaldehyde and 4% glutaraldehyde in the filtered seawater. No differences in preservation of the structures was observed between the fixation procedures and media. After 17 hours in fixation solution at 4 °C, 8 samples, one from each region and valve of the animals, were decalcified for 14 days in a solution containing 0.25 mol L^−1^ HEPES, 0.05 mol L^−1^EDTA and 1.0 v/v % glutaraldehyde stabilized at a pH of 8.0. All samples were washed three times with 0.1 M Na-cacodylate buffer (7.7 pH) and postfixed in the same buffer containing 1% OsO_4_ and 0.9% K_4_Fe(CN)_6_·3H_2_O for one hour. After washing with bi-distilled water, the samples were dehydrated in an ascending series of isopropanol solutions (30, 50, 70 and 90%), and contrasted with 2% uranyl acetate (in 100% ethanol) for 30 minutes, washed 3 times for 30 minutes each in 100% isopropanol and two times for 5 minutes in propyleneoxide and subsequently embedded in EPON resin.

#### High-pressure freezing and freeze substitution

*M. venosa* individuals no longer than 6 mm were dissected in culture seawater. With scalpels pieces of shell with the mantle epithelium attached were cut from the commissure and central region of dorsal and ventral valves. Samples were transferred to hexadecane and placed in aluminium planchets with an outer diameter of 3 mm and a 200 µm deep cavity, and covered with the flat side of planchets. Samples were then high pressure frozen with a Wohlwend HPF Compact 01 high-pressure freezer (Engineering Office M. Wohlwend GmbH) within 30 ms at a pressure of 2.3 × 10^8^ Pa. The planchet sandwiches were then opened and freeze substituted overnight in 0.2% OsO_4_, 0.1% uranyl acetate and 5% H_2_O in acetone ranging from −90 °C to 20 °C^75^. Samples were then embedded in EPON resin. Embedded samples were cut open using a diamond trimming knife (Diatome, Liechtenstein) and a Reichert Ultracut ultra microtome (Leica) to expose the mineralised shell.

#### Sample preparation for AFM imaging

For AFM imaging shell pieces of modern *Magellania venosa* shells were cut in longitudinal section from the umbo to the commissure and embedded in epoxy resin. Embedded sample surfaces were polished in 5 sequential mechanical steps down to a grain size of 1 µm. For the final step, etch-polishing was applied for three hours with a colloidal alumina suspension in a vibratory polisher. Subsequently, the samples were washed in Milli-Q water in an ultrasonic bath and rinsed with 80% ethanol.

#### Sample preparation for microstructure characterisation

For Electron Backscatter Diffraction (EBSD) analyses 5 × 5 mm pieces were cut out of the shell and embedded in epoxy resin. The surface of the embedded samples was subjected to several sequential mechanical grinding and polishing steps down to a grain size of 1 μm. The final step consisted of polishing with colloidal alumina (particle size ~0.06 μm) in a vibratory polisher. Finally, samples were coated with 4–6 nm of carbon.

## Methods

### Transmission Electron Microscopy (TEM)

Ultra-thin 60 nm sections were cut from chemically-fixed and decalcified samples using a diamond knife and the ultra-microtome. The sections were placed on carbon stabilized Formvar-coated copper hole grids and stained with 0.3% lead citrate. A Zeiss 912 TEM (Zeiss, Jena, Germany) equipped with an Omega energy filter, a goniometer stage and a 2k × 2k pixel camera (TRS, Moorenweis, Germany) was used to image the sections at 8000 times magnification with a 120 kV acceleration voltage using only elastically scattered electrons. To screen a large area of the outer mantle epithelium at high resolution up to 300 images were recorded at rectangular grids. The images were then aligned into large composite images using the TRS software. These composite images were used for structural and numerical analysis.

### Field Emission Scanning Electron Microscopy (FE-SEM)

Non-decalcified EPON resin embedded samples of high pressure frozen and freeze-substituted shell as well as chemically fixed shells, were knife polished by successively advancing the knife for 70, 40, 20, 10 and 5 nm 15 times for each step^76^. Samples were then mounted on aluminium holders using self-adhesive carbon pads and conductive glue and coated with 4 nm of carbon using a BAF 300 (BAL-TEC, Balzers, Liechtenstein). Samples were analysed with a Hitachi S5200 field emission scanning electron microscope (FE-SEM). For chemically fixed samples we used the secondary electron signal at 4 kV. To obtain material density contrast at high resolution for the high pressure frozen/freeze substituted samples, we used the converted backscattered electron signal to obtain so-called composite-rich images^77^ at 4 kV acceleration voltage and 20 µA emission current in analysis mode of the microscope. To test if the fibrous layer contained highly soluble mineral phases (e.g., an amorphous precursor phase of calcite) we first removed the 4 nm carbon layer using a diamond knife. The sample was then etched and organic material fixed simultaneously for 40 seconds using a 0.1 M HEPES (pH = 9.0) and 2.5% glutaraldehyde solution. Immediately after etching, the samples were dehydrated in 100% isopropanol 3 times for 10 seconds and were critical point dried in a BAL-TEC CPD 030 (Liechtenstein) device. The dried samples were coated with 3 nm platinum. Then, the same regions of the sample were imaged again in the Hitachi S5200 FE-SEM.

### Atomic Force Microscopy (AFM)

Samples were measured in contact mode with a JPK NanoWizard II AFM using silicon cantilevers. The measurements of height, lateral and vertical deflection traces were processed with the NanoWizard® IP image processing software and Gwyddion free and open source software. We used the “Gold” scale for colour. The height trace shows the surface height of the measured area while lateral and vertical deflection traces are the result of the interaction between the cantilever tip and the sample surface. With lateral deflection traces, we observed the different components within the shell (e.g., the organic membrane of the calcite fibres has a different interaction with the cantilever tip than the calcite of the fibres). We show all AFM results with vertical as well as lateral deflection trace measurements.

### Electron backscatter diffraction (EBSD)

EBSD measurements were carried out on a Hitachi SU5000 field emission SEM, equipped with an Oxford EBSD detector. The SEM was operated at 20 kV and measurements were indexed with the CHANNEL 5 HKL software. In this study information obtained from EBSD measurements is presented as band contrast measurement images. EBSD band contrast represents the signal strength of the EBSD-Kikuchi diffraction pattern in each measurement point and is displayed as a grey-scale component of EBSD scanning maps. The strength of the EBSD signal is high when a crystal is detected (bright), whereas it is weak or absent when a polymer is scanned (dark/black).

### Assessment of secreting and non-secreting OME portions

For distinguishing between secreting and non-secreting portions of the outer mantle epithelium (OME) we used several large TEM composite images from chemically fixed samples recorded at central shell regions. We measured the length of the outer mantle epithelium that is attached to the shell by apical hemidesmosomes and where two membranes could be observed such as at the apical membrane of the epithelial cells and the organic membrane at the proximal side of fibres (non-secreting parts of the epithelium). The length of these regions was compared, in perpendicular and longitudinal sections, with the length of those epithelium portions where the membrane lines the proximal side of the fibres as well as where apical hemidesmosomes are absent (secreting parts of the epithelium). For measurements, we used the open source software JMicroVision. The epithelial lengths were measured in basal parts of the epithelium where the cells are in contact with the basal lamina.

## Supplementary information

Calcite fibre formation in modern brachiopod shells - supplementary information

## Data Availability

The datasets generated during and/or analysed during the current study are available from the corresponding author on request.
