# Supplementary material for: Calcite fibre formation in modern brachiopod shells
Source: Sci Rep. 2019 Jan 24;9:598. doi: 10.1038/s41598-018-36959-z (PMC6345923; doi:10.1038/s41598-018-36959-z)
Supplement: Supplementary file 1 — Calcite fibre formation in modern brachiopod shells - supplementary information [file 41598_2018_36959_MOESM1_ESM.pdf]

Calcite fibre formation in modern brachiopod shells –

Supplementary information

Maria Simonet Roda[1], Erika Griesshaber[1], Andreas Ziegler[2], Ulrich Rupp[2], Xiaofei Yin[1], Daniela Henkel[3], Vreni Häussermann[4,5], Jürgen Laudien[6], Uwe Brand[7], Anton Eisenhauer[3], Antonio G. Checa[8,9], Wolfgang W. Schmahl[1]

[1]Department of Earth and Environmental Sciences, LMU, 80333 München, Germany
[2]Central Facility for Electron Microscopy, University of Ulm, 89069 Ulm, Germany
[3]Marine Biogeochemistry/Marine Systems, GEOMAR Helmholtz Centre for Ocean Research, 24148 Kiel, Germany
[4]Pontificia Universidad Católica de Valparaíso, Facultad de Recursos Naturales, Escuela de Ciencias del Mar, Avda. Brasil 2950, Valparaíso, Chile
[5]Huinay Scientific Field Station, Puerto Montt, Chile
[6]Alfred-Wegener-Institut Helmholtz-Zentrum für Polar- und Meeresforschung, 27568, Bremerhaven, Germany
[7]Department of Earth Sciences, Brock University, 1812 Sir Isaac Brock Way, St. Catharines, Ontario, L2S 3A1, Canada
[8]Departamento de Estratigrafía y Paleontología, Facultad de Ciencias Universidad de Granada, 18071 Granada, Spain
[9]Instituto Andaluz de Ciencias de la Tierra, CSIC-Universidad de Granada, 18100, Armilla, Spain.

Corresponding author: simonet@lrz.uni-muenchen.de

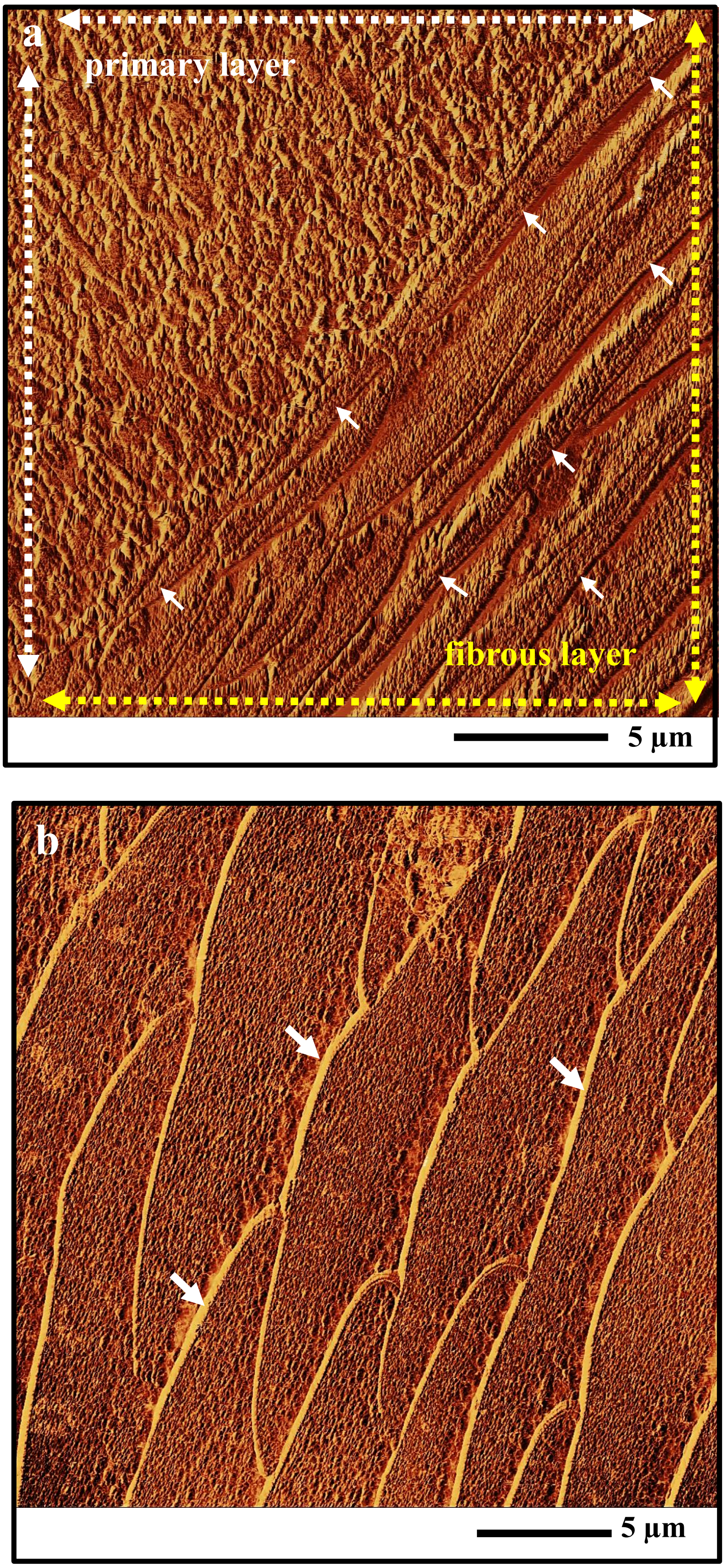

Figure A1. AFM lateral deflection images showing structural characteristics of the primary and the fibrous shell layers of the modern brachiopod *Magellania venosa*. Well visible is the smooth transition from the primary into the fibrous shell portion (a) as well as the organic membrane lining the proximal, convex surface of fibres (white arrows in a and b).

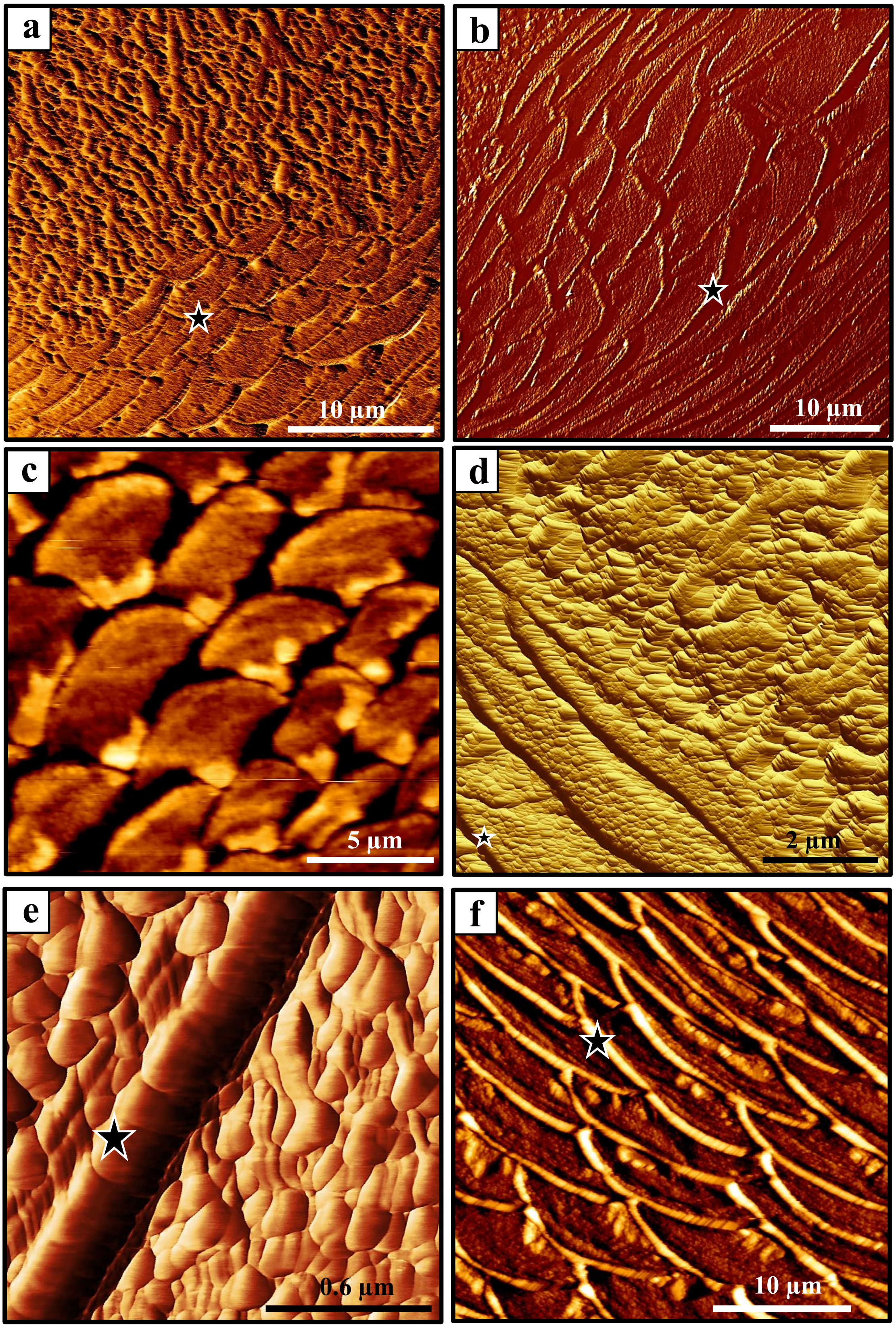

Figure A2. AFM lateral (a, b and d to f) and vertical (c) deflection images of the primary shell layer (a), the transition from the primary into the fibrous shell layer (a) and of stacks of fibres (a to f) within the fibrous shell layer. Corresponding vertical deflection images are shown in 1b, 2 and 3. The convex, proximal part of each fibre is always lined by an organic membrane (black star in a, b, d, e, f).

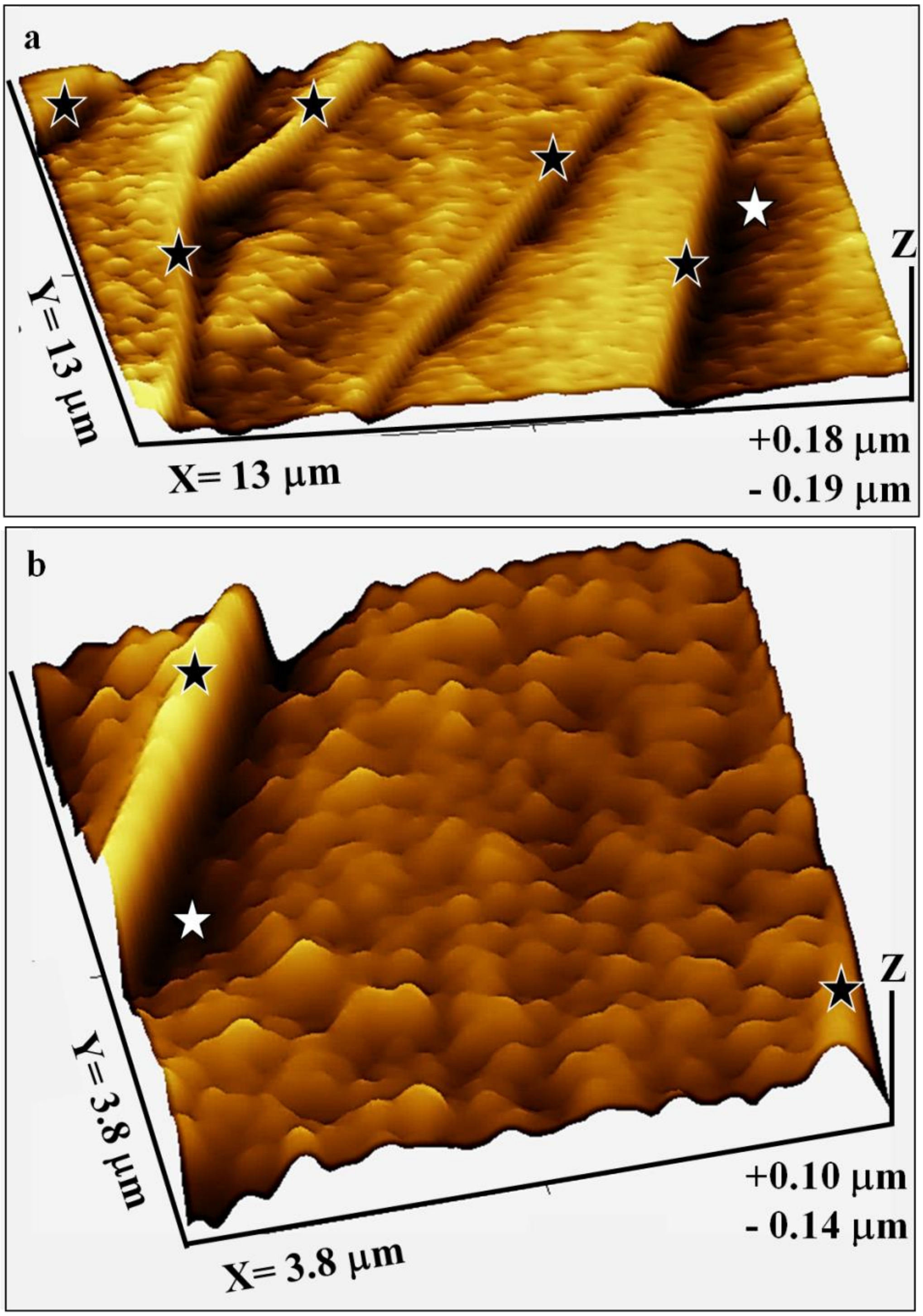

Figure A3. 3D representations of AFM height trace images of stacks of longitudinally cut calcite fibres. Well visible is the organic membrane lining at the proximal, convex part of a fibre (black stars in a, b), as well as the absence of any organic lining (membrane) along the apical portion of a fibre (white star in a, b). Instead, calcite fibres are not fully sheathed by an organic membrane, but are partially covered.

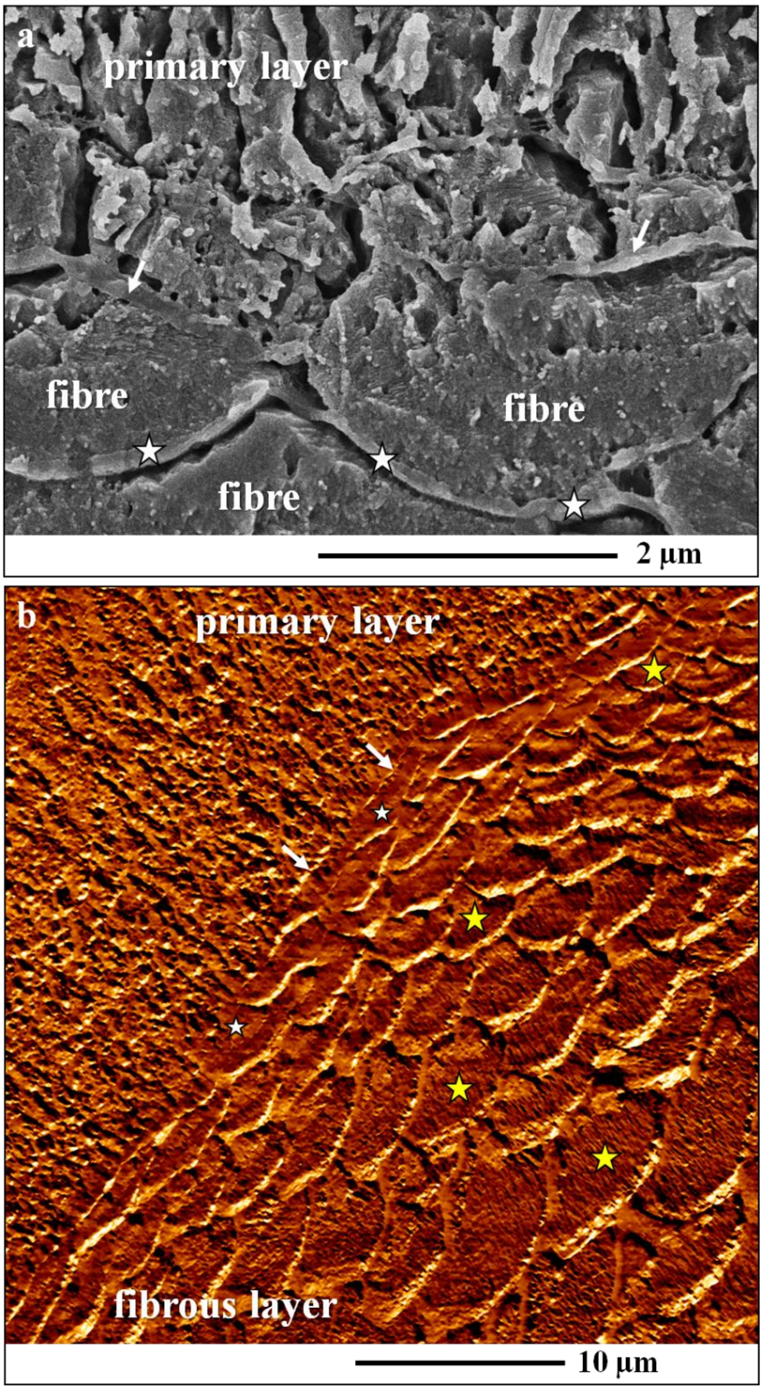

Figure A4. FE-SEM (a) and AFM lateral vertical deflection (b) images of primary and fibrous shell portions of *Magellania venosa*. The sample in (a) was microtome cut and polished, subsequently etched for 180 seconds at a pH of 6.5 and critical point dried. Both images depict the transition from the primary to the fibrous shell layer. We do not see a continuous membrane between the two shell layers; the transition from one layer into the other appears to be gradual. However, we see occasional inclusions of short sections of biopolymer membranes (white arrows in a and b) within the transitional zone from one shell layer to the other. White stars in (a) point to the proximal organic lining of the fibres. At the transition from the primary to the fibrous shell portion the shape of the fibres is not well developed (white stars in b). Mature fibres with their characteristic morphology and size appear a few rows away from the transition between the primary to the fibrous shell fabrics (yellow stars in b).

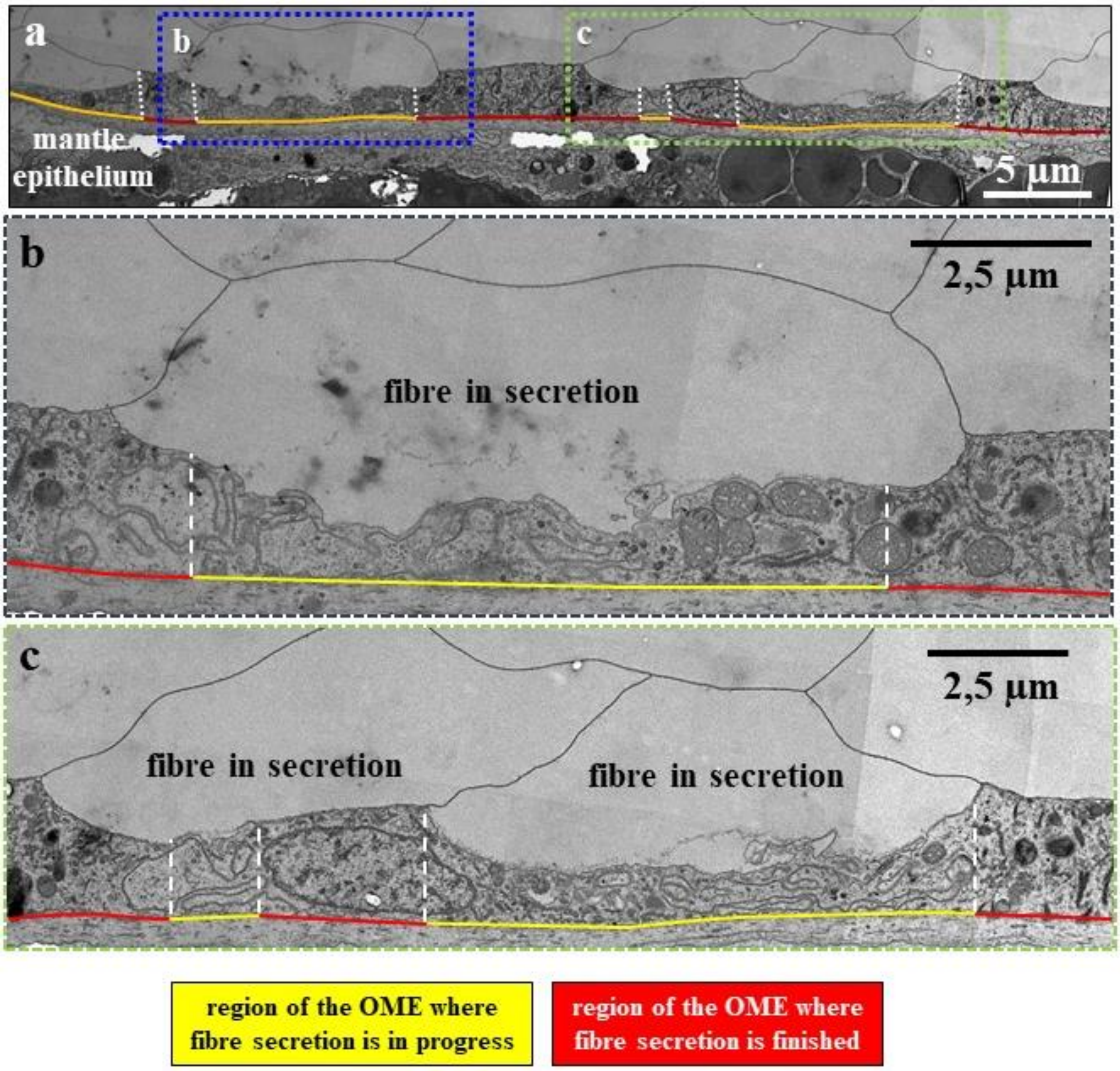

Figure A5. TEM micrographs of a chemically fixed shell sample from the ventral valve of *Magellania venosa*. **a**, **b**, **c**, Alternating shell portions where secretion is completed (indicated with red lines) with fibre portions that are in actively secreting (indicated with yellow lines). Mineral deposition ceases with secretion of the membrane lining at the proximal, convex side of the fibre.

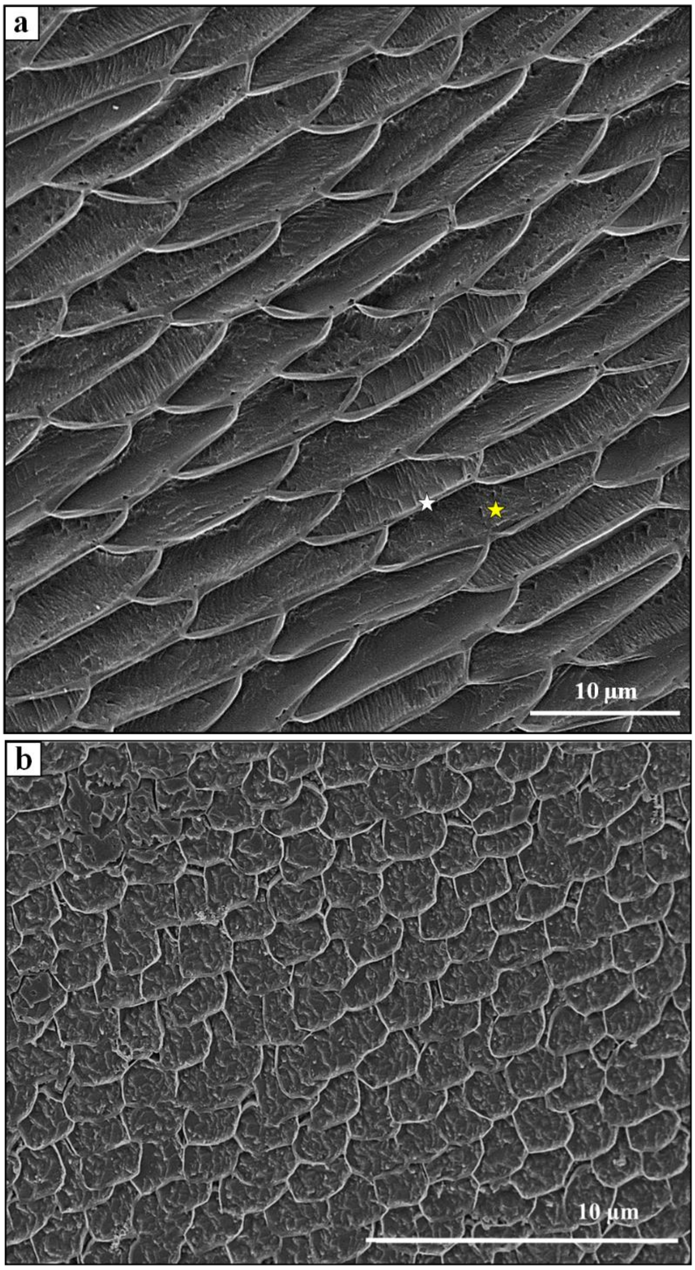

Figure A6. Mode of fibre assembly into stacks in the shell of the terebratulide brachiopod *Magellania venosa* (**a**), and in the calcitic shell portion of the bivalve *Mytilus edulis* (**b**). The samples were microtome cut and polished, subsequently etched for 180 seconds and critical point dried. Due to the concave-convex morphology of the brachiopod fibre and their interlocked packing into stacks, a biopolymer membrane (white star in a) is always present between two calcite fibres (yellow star in a). This gives the impression that a fibre is surrounded by an organic sheath, however, this is not the case. **b**, Cross-section through a stack of calcite fibres in the calcitic shell layer of *Mytilus edulis*. Fibre morphology, regularity in shape and extent of encasing of the calcite with an organic sheath is significantly different from that in the modern brachiopod *Magellania venosa*.

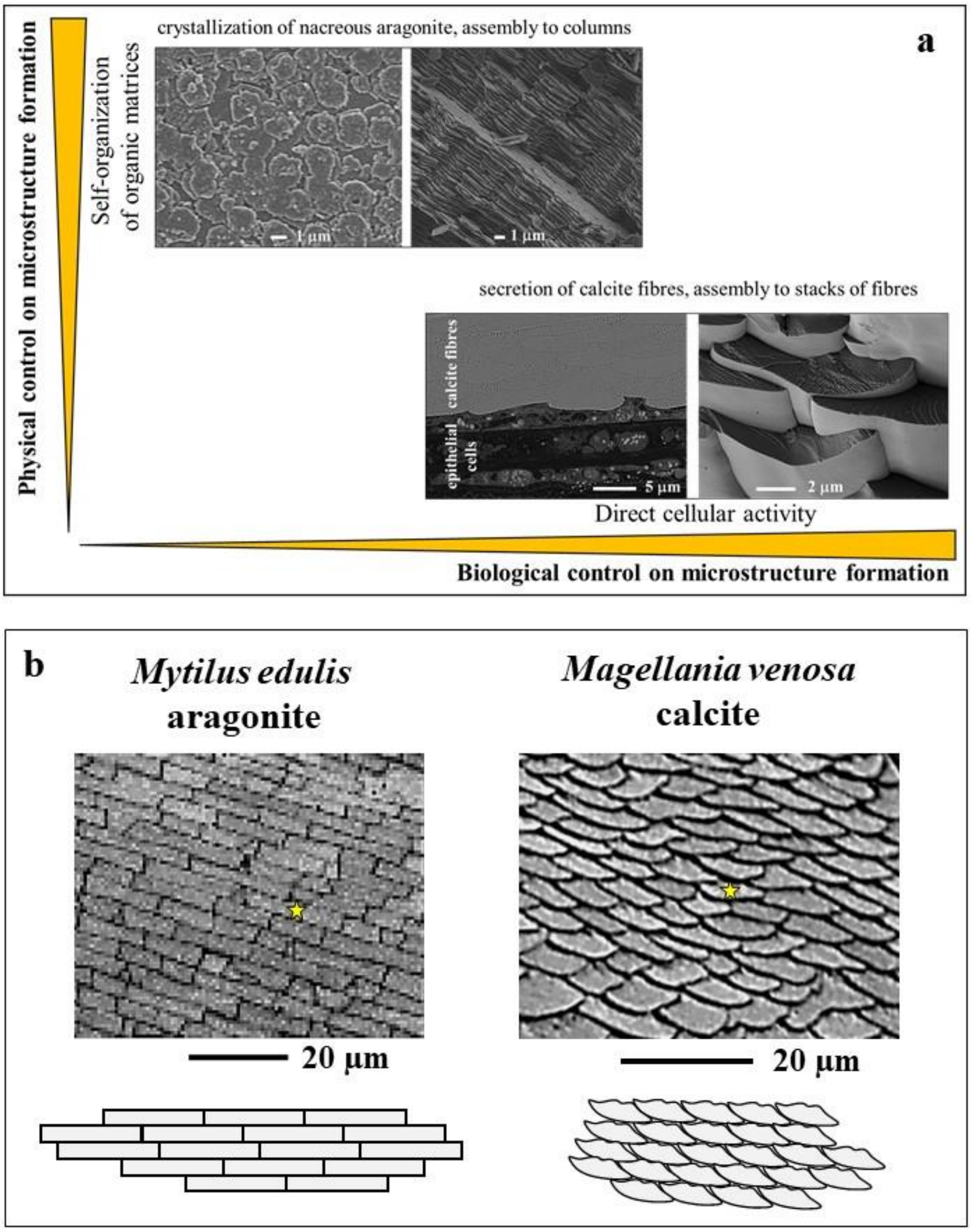

Figure A7. Processes involved in the fabrication of mollusc nacreous and brachiopod fibrous microstructures. Scheme in **(a)** is modified after Figure 10 in Checa et al. 2018. **a**, Whereas the nacreous aragonite mineral assembly takes place in self-organized organic matrices and the mineral unit, and shell microstructure formation is mainly controlled by physical determinants (Checa et al. 2018). Brachiopod shell microstructure formation is mainly biologically controlled as fibre secretion occurs at direct cellular contact. **b,** EBSD band contrast measurement image and corresponding schematic visualizing the mode of mineral unit assembly and interlocking in bivalve aragonite (*Mytilus edulis* nacreous tablets) and brachiopod calcite (*Magellania venosa* calcite fibres). A "brick-wall" arrangement of mineral units not only occurs in bivalve nacre, it is similar to the stacking mode of calcite fibres in the modern terebratulide brachiopod *Magellania venosa*. Yellow stars in (**b**) point to the basic mineral unit (tablet, fibre) that was chosen as a model unit for the schematics shown below the EBSD band contrast images.
